# Shadow-Based Vehicle Detection in Urban Traffic

**DOI:** 10.3390/s17050975

**Published:** 2017-04-27

**Authors:** Manuel Ibarra-Arenado, Tardi Tjahjadi, Juan Pérez-Oria, Sandra Robla-Gómez, Agustín Jiménez-Avello

**Affiliations:** 1 Control Engineering Group, University of Cantabria, Avda. Los Castros s/n, 39005 Santander, Spain; oria@teisa.unican.es (J.P.-O.); srobla@teisa.unican.es (S.R.-G.); 2 School of Engineering, University of Warwick, Gibbet Hill Road, Coventry CV4 7AL, UK; t.tjahjadi@warwick.ac.uk; 3 Department of Automatics, Electronic Engineering and Industrial Computing at the Polytechnic University of Madrid, 28006 Madrid, Spain; agustin.jimenez@upm.es

**Keywords:** driving assistance systems, forward collision avoidance systems, vehicle detection, shadow detection

## Abstract

Vehicle detection is a fundamental task in Forward Collision Avoiding Systems (FACS). Generally, vision-based vehicle detection methods consist of two stages: hypotheses generation and hypotheses verification. In this paper, we focus on the former, presenting a feature-based method for on-road vehicle detection in urban traffic. Hypotheses for vehicle candidates are generated according to the shadow under the vehicles by comparing pixel properties across the vertical intensity gradients caused by shadows on the road, and followed by intensity thresholding and morphological discrimination. Unlike methods that identify the shadow under a vehicle as a road region with intensity smaller than a coarse lower bound of the intensity for road, the thresholding strategy we propose determines a coarse upper bound of the intensity for shadow which reduces false positives rates. The experimental results are promising in terms of detection performance and robustness in day time under different weather conditions and cluttered scenarios to enable validation for the first stage of a complete FACS.

## 1. Introduction

Insufficient breaking distance is one of the leading causes of front-to-rear collisions in urban traffic. Forward Collision Avoidance Systems (FCAS) aid drivers to maintain a safe stopping distance relative to the vehicle ahead in order to avoid or at least reduce the number and severity of traffic accidents. A fundamental task of FCAS is vehicle detection which strongly influences the reliability of the system. Lately, vision-based vehicle detection systems are playing an important role in FCAS. Low cost cameras compared to other sensors such as LIDAR or RADAR, together with increasingly, powerful computers and advances in the fields of image processing and computer vision make vision-based systems a growing segment in FCAS.

Vision-based vehicle detection systems generally consist of two main stages [1]: hypotheses generation (HG) and hypotheses verification (HV). In the HG, regions in the image which potentially contain a vehicle are identified by a fast analysis throughout the image based on vehicle features. In the HV, hypotheses generated are further analysed (generally by a computationally-intensive machine learning method) to verify whether the candidates are vehicles. Since the output of the HG stage is the input of the HV, its reliability is important to ensure the detection of the image regions containing vehicles with minimum false candidates.

There are several factors that make hypotheses generation challenging. The size, shape and colour of a vehicle depend on its make and model, so the vehicle detection procedure cannot focus on a specific object. This together with changing scenarios, cluttered backgrounds and variable illumination contribute to make vehicle detection difficult. A cluttered background typical of urban traffic may cause apparent merging of background objects with the contour of the vehicle in the road scene captured by a video camera, whereas outdoor illumination which depends on the weather conditions, may modify the shape and colour of the vehicle ahead, resulting in poor vehicle detection.

Motivated by the aforementioned challenges, this paper focuses on the hypotheses generation, presenting a feature-based method to detect vehicles ahead in the target path. Hypotheses are generated according to the shadowed road region under the vehicles, which is a distinctive feature of a vehicle in both overcast and sunny conditions.

The shadow under the vehicle occurs due to the vehicle occluding the ambient light which comprises of skylight (on overcast days including cloudy and rainy conditions) or both skylight and sunlight (on sunny days). Due to the shape of a vehicle, the gap between the underside of the vehicle and the road surface is very small, thus occluding the road area under the vehicle from direct sunlight and some skylight, and exposing it to only a little amount of lateral skylight in both sunny and overcast conditions. This makes the road area very dark, with little texture and void of brightness. Even if the vehicle is travelling in the shade, the road area under the vehicle is darker than its surroundings which are illuminated by a higher amount of ambient light. Thus, as long as there is ambient light the shadow under a vehicle is present on the road, making it a reliable cue for vehicle detection in daytime.

In this paper we propose a novel strategy for the detection of the shadow which overcomes significant difficulties such as outdoors illumination as well as the presence of lateral shadows and traffic markings on the road. The method is designed to work in day time under different weather conditions in urban traffic, a challenging scenario characterized by cluttered backgrounds, and includes highways and extraurban roads. The proposed HG method is intended to integrate a complete vehicle detection system, i.e., HG followed by HV, to prevent front-to-rear collisions by detecting vehicles ahead in the target path.

The remainder of this paper is organised as follows. In Section 2, we review the related work. Section 3 presents the proposed HG method. Experimental results are presented in Section 4 and finally, Section 5 concludes the paper.

## 2. Related Work

Hypotheses generation methods can be classified into three categories [1]: stereo-based, motion-based and appearance-based. Stereo-based systems [2,3,4] involve finding correspondences between the left and right images of the stereo image pair of a scene which is a complex and time-consuming task. Motion-based methods exploit the optical flow of moving vehicles obtained by matching pixels from consecutives frames of an image sequence [5,6]. The computational cost of this method is expensive and requires the processing of several frames to detect a vehicle. Appearance-based methods are the most used approaches which exploit common vehicle features such as edges, symmetry, texture, colour, vehicle lights, shadows, etc. They are closely conditioned by illumination and cluttered backgrounds. Edges are one of the most used features in vehicle detection. Edge-based methods [7,8] build upon the rear view of a vehicle containing many horizontal and vertical structures, e.g., contour of the vehicle, license plate, rear window, bumper, etc. that cause high edge density in the image. Thus, a grouping of vertical and horizontal edges in the image has been used to determine a vehicle candidate. However, the background strongly influences the correct edge detection of the vehicle contour, causing merging of background objects with the contour of the vehicle. In addition, a cluttered background can present regions with similar edge density than the rear of the vehicle which may generate false positives. Symmetry-based methods [9,10] exploit the symmetry with respect to a vertical centreline of the vehicle rear. Vehicle candidates are determined by searching regions in the image with high horizontal symmetry. However the computation of symmetry is a time consuming procedure for real time applications. In addition, illumination can cause bright regions on the vehicle, generating a loss of symmetry and therefore a loss of true positives. Texture-based methods [11,12] assume the texture of vehicles is different from the texture of their surrounding road. The texture of the asphalt is generally very homgeneous whereas the texture of vehicles presents regions with a high intensity variation. However, this technique may generate a large quantity of false positives, especially in urban environment, where the background of the image may present elements with similar texture of vehicles. In colour-based methods [13,14], colour is used for segmenting the vehicle from the background. However, these methods are very sensitive to illumination changes and specular reflections that may cause the loss of true positives. Noting that taillights are an important feature for vehicle detection at night time, in [15,16] vehicle hypotheses are generated using a morphological filter to detect the taillight pair in a narrow horizontal search region. However, this approach is only applicable for night time vehicle detection.

As the hypotheses generation method proposed in this paper is a shadow-based vehicle detection method, we review its particular related work more thoroughly.

The shadow under a vehicle was first used for vehicle detection in [17] where intensity ranges of both the non-shadowed road and the region under the vehicle are established for sunny and overcast days. The shadow detection is reduced to a search for image regions whose intensity values are within the corresponding range. However, the intensity values differ for different types of asphalt, thus this method may be valid for a specific road only. A second attempt uses a horizontal edge detector applying brightness and correlation values constraints [18]. However the constraints and thresholding method used are not specified in [18].

The intensities of the shadow under a vehicle and road highly illuminated by ambient light depend on both asphalt and illumination (which is determined by the weather and time of day), thus the intensity threshold which separates them is not a fixed value and requires a thresholding strategy. In order to establish an intensity threshold, several approaches have assumed the shadow under vehicles is always darker than the surrounding road [17], and determined an approximate image grey level corresponding to the available (i.e., free) driving space in front of the ego-vehicle. Thus, regions in the image whose intensity is smaller than the grey level are expected to be the shadow under vehicles.

In [19] several masks generated by combining luminance and colour information are used to segment the image of a road scene into two classes: road and non-road. Dark areas with specific constraints (i.e., minimum size, height and width) on the road class are considered vehicle candidates. However, no thresholds either for the road or dark area detections are given in [19]. In [20] the free driving space is determined by the local image entropy method, and the shadow detection is via intensity thresholding, morphological filtering and region clustering stabilized over time. However, the grey level threshold separating road and the shadow under a vehicle is not provided in [20]. An alternative solution is proposed in [21] and later used in [22,23,24,25,26,27,28,29], where a coarse approximation of the free driving space is obtained by defining the lowest central region in the image delimited by edges. A normal distribution is assumed for the grey levels of the free driving space, and the shadow under a vehicle is defined as a region with intensity smaller than a threshold *m* − 3*σ*, where *m* and *σ* are respectively the mean and standard deviation of the grey levels of road pixels. This thresholding method has been demonstrated to be successful in certain scenarios (e.g., highways and extraurban roads) and weather conditions (e.g., overcast days). However, the method has some drawbacks when operating in urban traffic and sunny conditions. Firstly, the normal intensity distribution for the road is not always true because illumination may cause a non-uniform grey level variation. Thus even a well laid asphalted road can show zones where the intensity is significantly different. Moreover, the threshold *m* − 3*σ* is not the upper bound intensity of the shadow under a vehicle but it is a lower bound of the road intensity. This fact contributes to false positive detections as all elements darker than the road as well as lateral shadows are considered vehicle candidates. Furthermore, in urban traffic due to the permitted slower speed of vehicles the gap between a vehicle ahead and the ego-vehicle is narrower than in highways, and the boundary of the shadow under a close vehicle may appear in the image as a gradual intensity transition where edge detection (e.g., using Canny and Sobel operators) can easily fail to detect it. This may lead to inclusion of the shadow under the vehicle ahead as free driving space.

Lastly, [30] proposed a thresholding strategy based on the greyscale histogram assessment of only a region of interest (ROI) corresponding to a safety area (where collision is likely) in front of the ego-vehicle. When a vehicle is in the safety area, the grey level histogram of the ROI displays two intensity peaks. The lower peak corresponds to the shadow under a vehicle and the higher one to the road. Depending on the illumination both peaks undergo grey level variation, so the threshold is set according to the lower intensity peak as long as it is smaller than a fixed threshold. This approach demonstrates good vehicle detection rates under different illumination. However, the short safety distance between the two vehicles considered makes the method suitable only for urban traffic.

The method of detecting shadow under a vehicle proposed in this paper initially focuses on distinguishing the intensity transitions on the road due to shadow under the vehicle from those due to other elements by comparing pixel properties across them. From the set of upper pixels of the resulting transitions and based on the fact that the shadow under a vehicle is a very dark road region, a coarse intensity threshold is determined so that regions with intensity smaller than the threshold become candidates of the shadow. For each candidate, a refined intensity threshold is applied to reject adjacent transitions due to lateral shadows. Finally, after morphological filtering based on the knowledge of the pose and size of the vehicle, a ROI covering the frontal road area of the ego-vehicle is established. Only vehicles within the ROI are susceptible to a possible rear-end collision with the ego-vehicle, therefore only candidates within the ROI are considered the final vehicle hypotheses.

## 3. Hypotheses Generation Method

### 3.1. Searching Image Region

**Figure 1:**
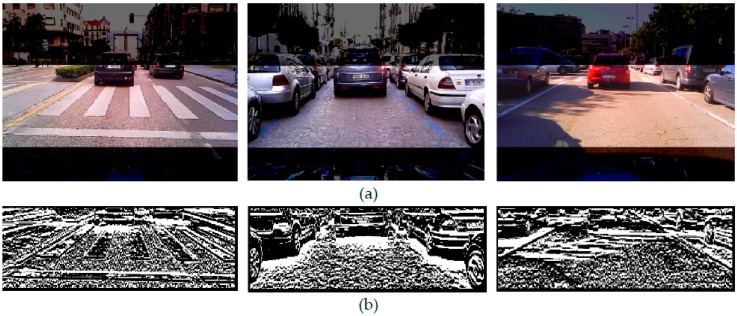
City centre roads with road markings, vehicles parked on both sides and cluttered bacgrounds in overcast and sunny conditions. (**a**) Searching image region (**b**) vertical intensity gradients *M*(*x*, *y*).

There are a wide variety of roads in a city, from narrow streets with a single lane to wide avenues with several lanes. In order to simplify a captured road scene, the search space in the incoming colour images is vertically shortened by using knowledge of the road scene perspective and assuming flat road surface. The camera is installed beside the vehicle’s rear-view mirror and the search area considered is a rectangular area covering the nearest road region ahead of the ego-vehicle, thus excluding image areas corresponding to distances not affecting the movement of the ego-vehicle (see Figure 1a). For our 240 × 320 camera the search area covers 110 × 320 pixels.

### 3.2. Vertical Intensity Gradients of Shadow

The detection of shadow under a vehicle ahead is initially based on the observation that shadows darken a road [17]. We extract the vertical intensity transitions from grey values of the road illuminated by ambient light to darker ones corresponding to the shadow (scanning the image bottom-up). Due to the pose of the vehicle ahead, the upper pixels of the transitions correspond to the shadow and the lower ones to the road which can be illuminated by skylight (on overcast days) or both skylight and sunlight (on sunny days). Prior to the intensity transitions extraction, the RGB colour image of the scene is converted to greyscale image *I*, and an averaging low pass filter with a 3 × 1 kernel is applied to reduce noise.

Depending on both illumination and gap distance, the intensity transitions due to the shadow under a vehicle can be sharp or gradual. Both direct sunlight and distant vehicles cause strong intensity transitions in the image, whereas skylight (diffuse) and close vehicles tend to cause smooth ones. In order to ensure the detection of both sharp and smooth transitions the process is carried out by performing a simple vertical intensity gradient operator with no threshold, i.e.,

(1) $$M(x,y)=\left\{ \begin{array}{l} 1,\;I(x,y)-I(x+1,y)<0\\ 0,\;\mathrm{otherwise}, \end{array}\right.$$

where *x* represents the rows and *y* the columns with respect to the top-left corner of the searched image region. The resulting binary mask *M*(*x*, *y*) includes gradients due to the shadow and to any other elements on the road such as asphalt noise, kerbs, lateral shadows, lane markings, etc. (see Figure 1b). In order to identify the gradients that correspond to the shadow, we exploit the spectral and intensity difference properties of the upper and lower pixels. The use of gradients instead of edges provides better results when comparing pixel properties across shadow boundaries. Unlike edges, gradients enclose the penumbra of shadows which is the transition region between the umbra and the road fully illuminated by ambient light. Thus, the upper pixels correspond to the shadow that falls onto the darker umbra, whereas the lower one falls onto the brighter road, avoiding pixels in penumbra. Hypotheses for shadow candidates are generated according to the following four conditions:

1. We exploit the property that the intensity of each of the red, green and blue components reflected off the road decreases across a shadow-road transition [31,32]. The analysis is performed for each gradient *i* of *M*(*x*, *y*). Denote the position of the upper and lower pixels of the intensity gradient *M_i_* as (*x_U_*, *y*) and (*x_L_*, *y*), respectively. Thus, the gradient *M_i_* becomes a candidate of the gradient of shadow if the intensity of the upper pixel {*R_i_*(*x_U_*, *y*), *G_i_*(*x_U_*, *y*), *B_i_*(*x_U_*, *y*)} is smaller than that of the lower {*R_i_*(*x_L_*, *y*), *G_i_*(*x_L_*, *y*), *B_i_*(*x_L_*, *y*)} for the three RGB colour channels. Otherwise *M_i_* is rejected, i.e.,

(2) $$M(\bar{x_{U}x_{L}},y)=\left\{ \begin{array}{l} 1,\;\;\;\;\;\;\;\;R_{i}(x_{U},y)<R_{i}(x_{L},y)\\ \;\;\;\mathrm{and}\;G_{i}(x_{U},y)<G_{i}(x_{L},y)\\ \;\;\;\mathrm{and}\;B_{i}(x_{U},y)<B_{i}(x_{L},y)\\ 0,\;\;\;\;\;\;\;\;\mathrm{otherwise}. \end{array}\right.$$

This constraint is effective for rejecting gradients caused by material changes and asphalt noise with small intensity difference between their upper and lower pixels.

2. We take into account the lack of light under a vehicle which makes the road region beneath it dark and colourless. The intensity is a linear combination of the *R*, *G* and *B* channels, thus the low intensity level of the road under the vehicle implies low levels of the three RGB components. In the Improved Hue Luminance Saturation (IHLS) space, saturation is defined as [33]

(3) S(x,y)=max[R(x,y),G(x,y),B(x,y)]−min[R(x,y),G(x,y),B(x,y)].

Accordingly, the saturation (the proximity of the three RGB components to a same value) decreases when the light illuminating the road changes from the total ambient light to a little amount of lateral skylight (road region under the vehicle). As the darkness under the vehicle increases the three RGB components decrease, reaching values close to the greyscale regardless of the colour of the asphalt. Thus, the perception of the road under the vehicle becomes close to the achromatic axis (*R ≈ G ≈ B*). Generally, asphalt roads are neutral surfaces so their saturation is low. In this case, the significant decrease in lighting under the vehicle makes the intensity difference between the RGB components smaller or equal to the road fully illuminated by ambient light. Thus, the gradient *M_i_* becomes a candidate of the gradient of shadow if the saturation of the upper pixel *S_i_*(*x_U_*, *y*) is smaller or equal to that of the lower *S_i_*(*x_L_*, *y*). Otherwise *M_i_* is rejected, i.e.,

(4) $$M(\bar{x_{U}x_{L}},y)=\left\{ \begin{array}{l} 1,\;S_{i}(x_{U},y)\leq S_{i}(x_{L},y)\\ 0,\;\mathrm{otherwise}. \end{array}\right.$$

In the IHLS space, the saturation avoids the normalization by brightness of colour spaces such as in HLS, HSI, HSV, etc. where the saturation involves division by intensity which is nearly null at low brightness. Dark colourless (achromatic) regions in the image reach normalized saturation values higher than the other more colourful (chromatic) regions. This problem is inherent to the normalisation within the achromatic zone [34].

3. Constraint is imposed based on the observation that the shadow under a vehicle is an achromatic region characterized by its low saturation whereas colourful (chromatic) regions are highly saturated. Thus, gradients with achromatic upper pixels become candidates of gradient of shadow.

Several approaches have focused on chromatic/achromatic pixel classification which is usually achieved by thresholding the pixel saturation and/or intensity with a fixed value. A first approach was proposed in [35] and later used in [32] where a pixel is considered achromatic if the sum of its RGB components is less than 30 on a range of 256. In [36] a pixel is classified as achromatic when its RGB components fall within the sphere of radius 4*σ_N_* centred at the origin of the RGB space, where *σ_N_* is the standard deviation of the sensor noise at low illumination. In [37,38] a pixel is considered achromatic if its intensity is below 10 or above 90, or if its normalized saturation is under 10, where the saturation and intensity values are normalized from 0 to 100. Finally, in [39] pixels are classified as achromatic if their normalized saturation is below 20% of the maximum saturation.

The aim of the chromatic/achromatic pixel classification is to reject gradients with colourful upper pixels which do not clearly correspond to the shadow underneath a vehicle. Therefore we propose a coarse chromatic/achromatic pixel classification where a pixel is categorized as chromatic if its IHLS saturation is higher than 25% of the maximun saturation i.e., 64 on a range of 256. This coarse threshold was empirically established from a wide set of test images acquired on different asphalts and weather, being very conservative at low intensity ensuring the classification of shadow pixels as achromatic. However, as the intensity increases the threshold is less conservative, making it useful to reject upper pixels of gradients onto colourful objects such as vehicles or elements on the pavement.

Thus, gradient *M_i_* becomes a candidate of the gradient of shadow if the IHLS saturation of the upper pixel *S_i_*(*x_U_*, *y*) is smaller or equal to 64. Otherwise, *M_i_* is rejected, i.e.,

(5) $$M(\bar{x_{U}x_{L}},y)=\left\{ \begin{array}{l} 1,\;S_{i}(x_{U},y)\leq 64\\ 0,\;\mathrm{otherwise}. \end{array}\right.$$

4. Finally, a constraint based on the intensity difference between the upper and lower pixels of the gradients is proposed. Depending on the ambient illumination and type of asphalt, the intensities of the road and the shadow vary, however their difference is significant even if the road is in the shade. The intensity of the light reflected off a surface is the product of incident light and surface reflectance [40]. Thus, on a sunny day the intensity of the reflected light *I_road_*(*λ*, *p*) at a point *p* on the road for both sunlight *I_sun_*(*λ*, *p*) and skylight *I_sky_*(*λ*, *p*), and for some viewing geometry is [41]

(6) $$I_{road}(\lambda ,p)=(I_{sun}(\lambda ,p)+I_{sky}(\lambda ,p))\cdot \rho_{road}(\lambda ,p),$$

where *λ* is the wavelength and *ρ_road_*(*λ*, *p*) is the reflectance of the road. In both overcast condition and road in the shade, the ambient illumination is composed only of skylight, thus the reflected light *I_road_*(*λ*, *p*) at a point *p* on the road becomes

(7) $$I_{road}(\lambda ,p)=I_{sky}(\lambda ,p)\cdot \rho_{road}(\lambda ,p).$$

On the other hand, the road region under the vehicle is illuminated by a little amount of lateral skylight. The reflected light at a point *p* on the road under the vehicle *I_shadow_*(*λ*, *p*) in the three possible cases, i.e., sunny and overcast conditions as well as on road in the shade, is

(8) $$I_{shadow}(\lambda ,p)=\delta \cdot I_{sky}(\lambda ,p)\cdot \rho_{road}(\lambda ,p),$$

where *δ* is a fraction of 1 denoting the amount of skylight reflected off the road under the vehicle and depends on the height between the underside of the vehicle and the ground. As the reflectance of the road is constant, the intensity difference between a point on the road illuminated by ambient light and a point on the road region under the vehicle for both overcast condition and road in the shade is

(9) $$I_{road}(\lambda ,p)-I_{shadow}(\lambda ,p)=(1-\delta )\cdot I_{sky}(\lambda ,p)\cdot \rho_{road}(\lambda ,p)=(1-\delta )\cdot I_{road}(\lambda ,p).$$

The geometric factor *δ* is small so (1 − *δ*) is large, causing a strong intensity difference between the road fully illuminated by skylight and the shadowed road under the vehicle. On a sunny day, the road is in addition illuminated by sunlight which makes the intensity difference even stronger. However, it is very difficult to obtain an accurate value of *δ* since the height between the underside of the vehicle and the ground depends on the vehicle make and model. Therefore, a coarse factor *δ* is considered not to accurately identify gradients due to shadow but reject gradients whose intensity difference do not clearly correspond with the significant intensity difference across the former. From the analysis of a set of different kind of vehicles (i.e., cars and vans) we propose a coarse factor *δ* of 0.5 so the amount of skylight reflected off the road under the vehicle is considered 50% of ambient light. Hence the intensity difference between the upper and lower pixels of the gradient due to the shadow under a vehicle satisfies

(10) $$I_{road}(\lambda ,p)-I_{shadow}(\lambda ,p)\geq (1-\delta )\cdot I_{road}(\lambda ,p),$$

where simplifying and replacing *δ* by 0.5 gives

(11) $$I_{shadow}(\lambda ,p)\leq \delta \cdot I_{road}(\lambda ,p)\Rightarrow \frac{I_{shadow}(\lambda ,p)}{I_{road}(\lambda ,p)}\leq 0.5.$$

Thus the gradient *M_i_* becomes a candidate of the gradient of shadow if the relationship between the upper *I_i_*(*x_U_*, *y*) and lower *I_i_*(*x_L_*, *y*) pixels is lower or equal to 0.5. Otherwise, *M_i_* is rejected, i.e.,

(12) M(xUxL¯,y)={1, Ii(xU,y)Ii(xL,y)≤0.5 0, otherwise.

Using a wide range of test images captured in shadowed and non-shadowed roads as well as different types of vehicles ahead, we verified that the geometric factor *δ* is very conservative (the illumination under the vehicle is quite lower than 50% of ambient light), ensuring the correct classification of gradients due to shadow and contributing to the rejection of gradients due to soft lateral shadows, asphalt noise, elements on the pavement, etc.

**Figure 2:**
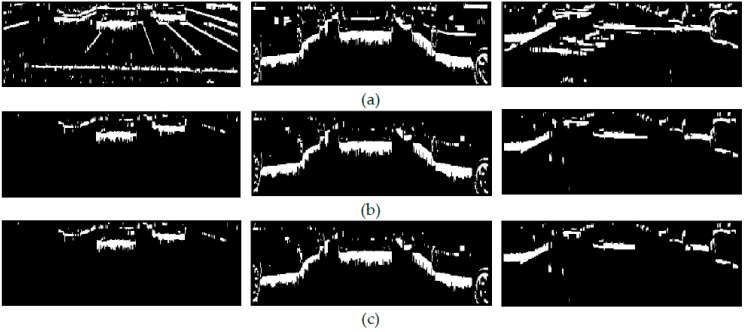
(**a**) Binary masks *M*(*x*, *y*) of Figure 1 after application of the saturation and intensity difference constraints, (**b**) *M*(*x*, *y*) after intensity thresholding and (**c**) *M*(*x*, *y*) after extraction of adjacent gradients due to lateral shadows.

Figure 2a show the resulting binary mask *M*(*x*, *y*) after application of the saturation and intensity difference constraints, i.e., Equations (2), (4), (5) and (12). It can be observed that gradients due to the shadow under vehicles satisfy the conditions whereas most of the gradients caused by other elements in the scene are rejected from *M*(*x*, *y*). Nevertheless, the gradients due to colourless elements such as lane markings, lateral shadows, kerbs and noisy elements still remain. In order to identify the gradients due to shadow, intensity thresholding is performed.

### 3.3. Intensity Threshold for Shadow

Unlike thresholding methods that determine a threshold which is a coarse lower bound of the intensity for road [21], the thresholding strategy we propose determines a coarse upper bound of the intensity for shadow under a vehicle from the upper pixels of the gradients remaining in the binary mask *M*(*x*, *y*) after application of the saturation and intensity difference constraints.

The upper pixels of the gradients remaining in *M*(*x*, *y*) correspond to the darkest pixels of the shadow, kerbs, asphalt noise, lateral shadows, lane markings, oil stains, etc. However, two observations can be made:

1. The shadow is darker than the road illuminated by ambient light, and thus darker than the upper pixels of the gradients due to lane markings, asphalt noise and lateral shadows.
2. The shadow is generally darker than any asphalt stain [17,21,30] and kerb (where the vertical side of a kerb is shadowed owing to the occlusion of a half hemisphere of skylight).

Therefore, of all the upper pixels of the remaining gradients in *M*(*x*, *y*), those corresponding to shadow under a vehicle are generally the darkest. Hence, the mean intensity value *m* of the set composed of the upper pixels of all the gradients in *M*(*x*, *y*) is a coarse upper bound for the shadow under a vehicle, i.e.,

(13) $$m=\frac{1}{n_{p}}\cdot \sum_{i=1}^{n_{p}}I_{i}(x_{U},y),$$

where *n_p_* is the total number of upper pixels of the gradients. Thus, gradients whose upper pixel intensity is lower that *m* become candidates of gradient due to shadow.

Nevertheless, in road scenes without gradients whose upper pixels have high intensity values (corresponding to lane markings, lateral shadows, etc.), the mean intensity of the pixels is not a reliable upper bound for the shadow. Let us consider a binary mask *M*(*x*, *y*) of a road scene where there is only the cluster of gradients due to shadow under a vehicle. In this case, the upper intensity bound is given by the largest intensity value of the upper pixels of the cluster so the mean value *m* would be an incorrect threshold for shadow. In this case no intensity thresholding is required.

In order to determine if *M*(*x*, *y*) includes gradients due to elements whose upper pixels present high intensity, we compute the standard deviation *σ* of the set of upper pixels, which indicates the data dispersion with respect to the mean value *m*. We consider the case of small standard deviation which denotes gradients where the intensities of the upper pixels are close to the mean, and apply the intensity threshold *m* if the standard deviation is greater than one third of the mean value, i.e., *σ* > *m*/3. Otherwise, no intensity threshold is applied.

The gradient *M_i_* becomes a candidate of gradient of shadow for low standard deviation or for high standard deviation if the intensity of its upper pixel *I_i_*(*x_U_*, *y*) is smaller than the mean intensity value *m* of the set. Otherwise, *M_i_* is rejected, i.e.,

(14) $$M_{i}(\bar{x_{U}x_{L}},y)=\left\{ \begin{array}{l} 1,\;\;\;\;\;\;\sigma \leq \frac{m}{3}\\ \;\;\;\mathrm{or}\;(\sigma >\frac{m}{3}\;\mathrm{and}\;I_{i}(x_{U},y)<m)\\ 0,\;\;\;\;\;\;\mathrm{otherwise}, \end{array}\right.$$

where

(15) $$\sigma =\sqrt{\frac{1}{n_{p}}\cdot \sum_{i=1}^{n_{p}}{\left(I_{i}(x_{U},y)-m\right)}^{2}}.$$

Figure 2b show the effectiveness of the mean value as intensity threshold for rejecting gradients due to lane markings, asphalt noise and most lateral shadows. On overcast days, vehicles do not cast lateral shadows thus the intensity thresholding usually leads to the retention of clusters of gradients composed only of those corresponding to shadows under vehicles (see Figure 2b left and center). However, on sunny days some gradients corresponding to dark lateral shadows adjacent to the gradients due to shadow under a vehicle may satisfy the intensity threshold and thus remain in *M*(*x*, *y*) (see Figure 2b right). In order to identify them, a further refined intensity threshold is applied to each resulting cluster. The binary mask *M*(*x*, *y*) of the road scene in Figure 2a center is basically composed of clusters of gradients due to shadow under a vehicle thus obtaining a low standard deviation value, *σ* < *m*/3. In this case, no intensity thresholding is applied (see Figure 2b center).

**Figure 3:**
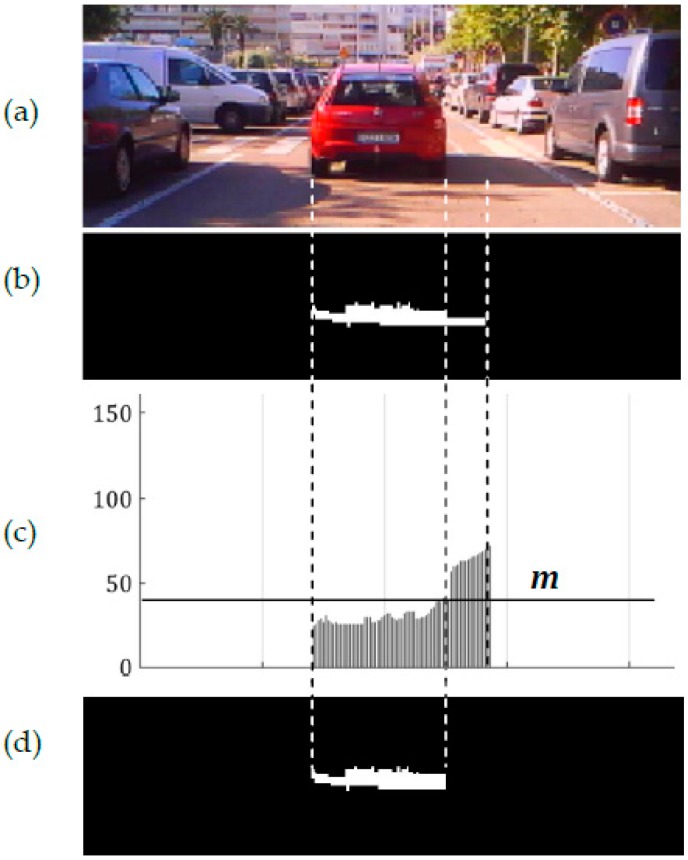
Intensity thresholding of the cluster of gradients under a vehicle in Figure 2 right. (**a**) Incoming image; (**b**) cluster of gradients due to both shadow under the vehicle and lateral shadow; (**c**) intensity of upper pixels of the cluster; and (**d**) resulting cluster after refined intensity thresholding.

In a cluster composed of gradients due to both shadow under a vehicle and a lateral shadow (see Figure 2b right and Figure 3b, the intensities of the upper pixels of the former are very similar to each other and significantly smaller than those of the latter which are illuminated by a higher amount of skylight (see Figure 3c). 

The standard deviation of the set of upper pixels of the gradients comprising the cluster is of high value whereas that of a cluster comprising only of gradients due to shadow under a vehicle is small. Therefore, the standard deviation *σ* (i.e., Equation (15)) is computed and for gradient values smaller than one third of the mean value, no adjacent gradient due to a lateral shadow is considered, and no threshold is applied to the cluster. Otherwise, an intensity threshold is computed using Equation (13), where in this case, *n_p_* is the number of upper pixels of the cluster under evaluation. Gradients of the cluster whose upper pixels are greater than the intensity threshold are rejected as gradients due to shadow under a vehicle.

Figure 2c right and Figure 3d show the resulting binary mask *M*(*x*, *y*) after thresholding and rejecting adjacent gradients due to the lateral shadow cast by the vehicle. Clusters composed of gradients due to shadows under the vehicles in Figure 2b left and center do not include adjacent gradients due to lateral shadows, giving small standard deviation values and thus they are not thresholded (see Figure 2c left and center).

### 3.4. Morphological Filter and Region of Interest

**Figure 4:**
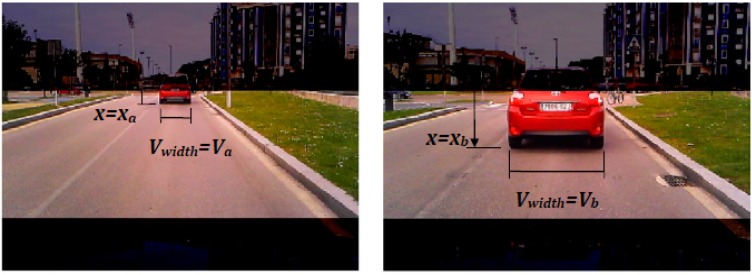
Variation of the width of a vehicle according to the vehicle vertical location in the image.

After intensity thresholding, a morphological filter based on the knowledge of the pose and width of the vehicle in the image is applied to obtain the final vehicle hypotheses. From the rear view of the vehicle ahead, the upper edge of the cluster of gradients due to the shadow under a vehicle is horizontal and its width matches with the width of the vehicle. Thus the width of the clusters of gradients in the binary mask *M*(*x*, *y*) is compared to that of a vehicle. Nevertheless, the width of a vehicle varies slightly depending on the make and model, thus an ideal vehicle width is assumed equal to the width of the ego-vehicle. Due to perspective projection, the width (in pixels) of the vehicle ahead varies linearly with respect to its vertical location *x* (in pixels) in the image (as illustrated in Figure 4). 

This relationship is determined by two frames of an image sequence where the vehicle ahead is at different distances away from the ego-vehicle. For our camera setting, in the first frame the vehicle ideal width *v_a_* is 30 pixels and the bottom of the vehicle *x_a_* is located at 15 *x*-coordinate, whereas in the second frame the vehicle ideal width *v_b_* is 178 pixels and its bottom *x_b_* is located at 100 *x*-coordinate (where *x*-coordinate represents the row with respect to the top of the searched image region). Thus, the linear relationship between the ideal width *v_width_* of the vehicle ahead and its vertical location *x* in the image is

(16) $$\frac{v_{width}-v_{a}}{v_{a}-v_{b}}=\frac{x-x_{a}}{x_{a}-x_{b}}\Rightarrow v_{width}=3.9+1.74\cdot x.$$

This relationship is specific to the resolution of the image as well as to the elevation and tilt of the camera installed in the ego-vehicle.

**Figure 5:**
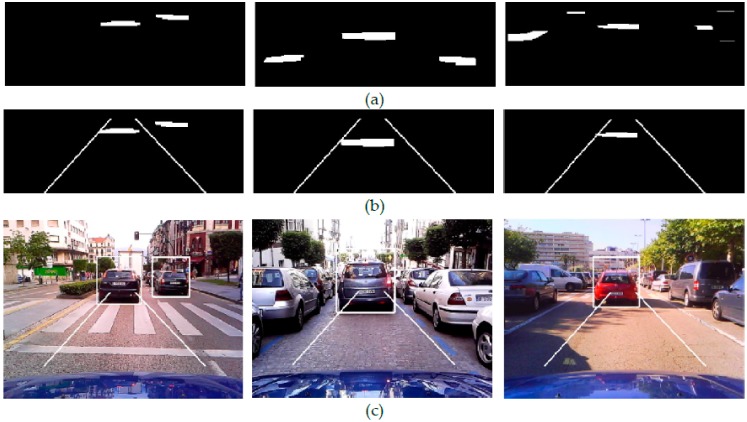
(**a**) Binary mask *M*(*x*, *y*) of Figure 2 after horizontal clusters extraction (**b**) *M*(*x*, *y*) after morphological thresholding and ROI establishment; and (**c**) vehicle hypotheses including those out of the ROI.

The filtering is as follows. First, horizontal clusters in *M*(*x*, *y*) are extracted by an opening operation using a structuring element based on the minimun ideal vehicle width, i.e., the width of the vehicle placed furthest. The proposed system is intended for urban traffic and it is designed to detect vehicles at a distance up to 20 m from the ego-vehicle. Thus the size of the structuring element corresponds to the width of the vehicle at 20 m which is obtained experimentally by placing a vehicle at this distance. This morphological operation focuses on eliminating vertical and inclined parts of clusters in *M*(*x*, *y*) such as those corresponding to kerbs and lateral sides of both a parked vehicle and a vehicle travelling in parallel lanes (see Figure 5a).

Second, a size filter is applied. Clusters of *M*(*x*, *y*) whose width is larger than 0.8·*v_width_* and shorter than 1.2·*v_width_* at the vertical location are finally considered candidates of gradients of shadow under a vehicle (see Figure 5b). To compute the vertical location of a cluster we consider the vertical location of its upper pixels which correspond with the bottom part of the vehicle.

After size filtering, for each candidate a bounding box containing the vehicle hypothesis is generated. In order to correctly frame the rear of the vehicle, the width of the cluster is horizontally lengthened by 5% of its width to both the right and left. To encompass all kinds of vehicles, i.e., cars and vans, a standard aspect ratio of the rear of the vehicle is assumed as in [30] where based on a set of hypotheses containing different vehicle models the height of the box is set equal to 130% of its width, ensuring the correct frame of tall vehicles, i.e., vans. Bounding boxes containing vehicle hypotheses are shown in Figure 5c.

Finally, a ROI is established focusing on the area at risk of a rear-end collision (see Figure 5b,c). The ROI is considered to comprise of a safety area, i.e., the stretch of the road up to 20 m in front of the ego-vehicle (15 *x*-coordinate) with a width equal to the width of the ego-vehicle. Only vehicles within the safety area are susceptible to a rear-end collision, thus only these vehicles are the target of the system. In this way, candidates detected within the ROI, either in whole or in part, are considered the final vehicle hypotheses (see Figure 5c).

## 4. Experimental Results

Experiments were carried out on image sequences acquired using an onboard camera which provided 240 × 320 colour frames with an 8-bit pixel depth. A total of 13,200 road images were captured in real traffic under sunny and cloudy conditions. The data consists of a large variety of urban traffic scenes composed of narrow and wide roads. We also used the publicly available Caltech dataset [42,43] for driving assistance systems which consists of 526 road frames of 240 × 360. In the Caltech dataset the image resolution, elevation and tilt of the camera in the ego-vehicle differ from those of our dataset, thus parameters of the searched image region, morphological filter and ROI are adapted. The searched area considered for Caltech dataset is an image region of 130 × 360 pixels covering the road region ahead of the ego-vehicle. The relationship between the width of the vehicle ahead and its vertical location in the image is determined by two frames of an image sequence of the dataset where a same vehicle ahead is at different distances away from the ego-vehicle and its width is assumed the ideal vehicle width, obtaining *v_a_* = 80, *x_a_* = 22, *v_b_* = 240, *x_b_* = 115, and from Equation (16), *v_width_* = 42.1 + 1.72*x*, where *x* is its vertical location. Finally, it is not possible to determine the vertical location in the image corresponding to 20 m away, thus the ROI is considered to comprise of the searched image region with a width equal to the ideal vehicle width.

**Figure 6:**
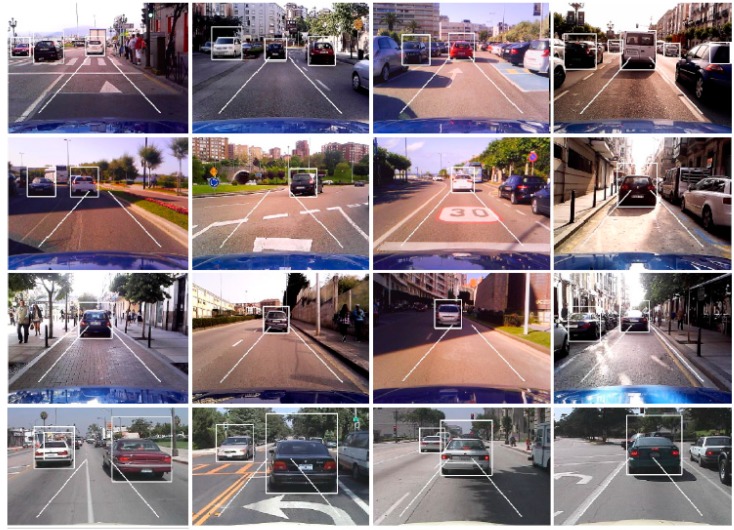
Example results of the hypotheses generation.

Figure 6 shows some example results of the HG stage in scenes of our dataset (top and middle rows) and the Caltech dataset (bottom row) where in order to better show the performance of the method, the hypotheses generated both inside and outside of the ROI are illustrated.

The method demonstrates high reliability as it correctly detects the clusters of gradients due to shadows under the different lighting conditions. The proposed thresholding strategy makes the method robust to lateral shadows and traffic markings on the road, minimizing the number of missed vehicles and false detections. 

A remarkable feature of the method is its ability to correctly frame vehicle hypotheses on sunny days in which the sun is in front of the ego-vehicle. In this situation, the vehicle ahead casts a rear lateral shadow (see right column of Figure 6) which makes difficult the correct framing of the vehicle’s rear when shadow boundaries are hypothesized by edges as in [21]. However, the proposed method exploits gradients which enclose the total intensity transition from the brightest road region to the darkest one which corresponds to the bottom of the vehicle, and thus result in a more accurate framing of the rear of the vehicle ahead.

**Figure 7:**
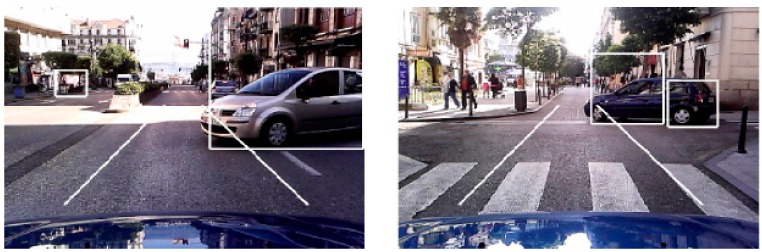
Hypotheses generated by vehicles travelling transversely on crossroads.

On the other hand, the use of shadow under vehicles may lead to hypotheses of vehicles which are out of the scope of the system such as vehicles travelling in parallel lanes (in both directions), vehicles parked by the lane or vehicles whose rear is occluded to some extent (generally by other vehicles). However, as the system focuses on vehicles travelling within the ROI, the number of final vehicle hypotheses is significantly reduced. Special mention must be made of vehicles travelling transversely to the ego-vehicle trajectory, i.e., in crossroads (see Figure 7) and roundabouts. The clusters of gradients due to their shadows can satisfy the morphological filter, thus providing hypotheses of vehicles out of the scope of the system.

The use of shadow under vehicles makes the method limited to day time under natural illumination conditions. Most of the vehicle features commonly used for vehicle detection in day time, i.e., edges, corners, texture, shadows, etc. are difficult or impossible to detect in darkness or night time [15], thus vision-based vehicle detection systems for night time are ad-hoc systems that are limited to the night time lighting conditions.

Artificial illumination, i.e., used during night time and in tunnels, is direct light which depending on the light source location relative to the vehicle, may cause shadow under vehicles (e.g., for light source on ceiling) or outside the vehicles’ vertical projection on the ground (e.g., for light source close to ground level). Thus the presence of the shadow under a vehicle at night or in a tunnel is not assured, making the vehicle detection method unreliable. Night time and in tunnels are scenarios that are outside the scope of the proposed method.

**Figure 8:**
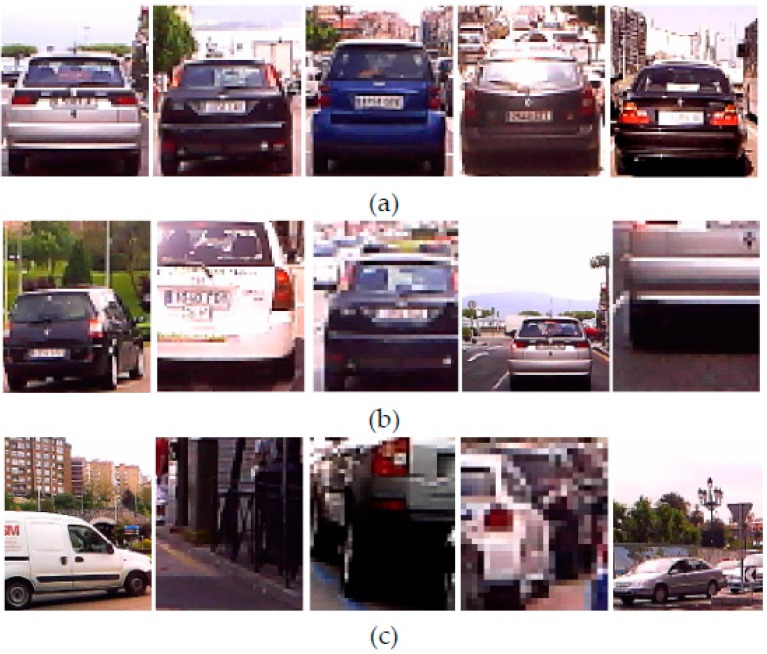
Example results: (**a**) positive hypotheses, (**b**) hypotheses corresponing to vehicles incorrectly framed; and (**c**) false positives corresponding to non-vehicles or vehicles out of system’s scope.

Quantitative results of the HG are presented in Table 1 where a hypothesis is considered positive *P* if the rear of the vehicle is correctly framed (see Figure 8a).

Hypotheses corresponding to vehicles whose rears are incorrectly framed *FNVIF* (see Figure 8b) are included as false positives *FP* together with missed vehicles *FNVM*. The detection rates *PR* and *FPR* are defined as

(17) $$PR(\%)=\frac{P}{V}\cdot 100,\;FPR(\%)=\frac{FP}{H}\cdot 100,$$

where *V* is the total number of vehicles within ROI, and *H* is total number of hypotheses generated.

The results show high rates in positive detection *PR*, achieving 98.04% and 97.71% on cloudy and sunny conditions, respectively. The loss in positives detection rate is not mainly due to vehicles missed but vehicles detected which are incorrectly framed. In both weather conditions, the number of missed vehicles is very low which demonstrates the HG method is very reliable. The number of hypotheses containing a non-vehicle *FP* (see Figure 8c) is relatively low for a HG stage, achieving rates *FPR* of 6.79% and 8.08% for cloudy and sunny conditions, respectively.

The morphological filter and the consideration only of hypotheses within the ROI strongly contributes to the low rate of FP.

Finally, the performance of the HG method is compared to the well known method in [21] where the shadow under a vehicle is defined as the upper region of edges with intensity smaller than the threshold *m* − 3*σ*, where *m* and *σ* are the mean and standard deviation of the free driving road delimited by edges (extracted using Sobel operator). After morphological filtering, edges due to adjacent lateral shadows are removed. Since the specifications of the morphological filter, the lateral shadows removal method and experimental results are not given in [21], a quantitative comparison is not possible. Therefore, we focus on comparing the intensity threshold *Th*_1_ proposed in [21] and *Th*_2_ proposed in this paper by means of two examples. The comparison of both thresholds is very indicative of the performance of the two methods being compared. This is because the result of the intensity thresholding is a binary mask where only the shadows under vehicles are supposed to remain. An ideal intensity thresholding will provide a binary mask where only pixels corresponding to shadow under vehicles remain. No lateral shadow removal or morphological filter should be necessary. Thus, the more accurate the threshold is, the better is the detection of the shadow under vehicles. A method which establishes a very conservative threshold will include as shadow other elements of the image that are not shadow, thus providing false positives. Conversely, as the accuracy in the threshold increases, fewer elements of the image which are not shadow will be included as shadows, therefore the method will be better as it will generate fewer false positives.

**Figure 9:**
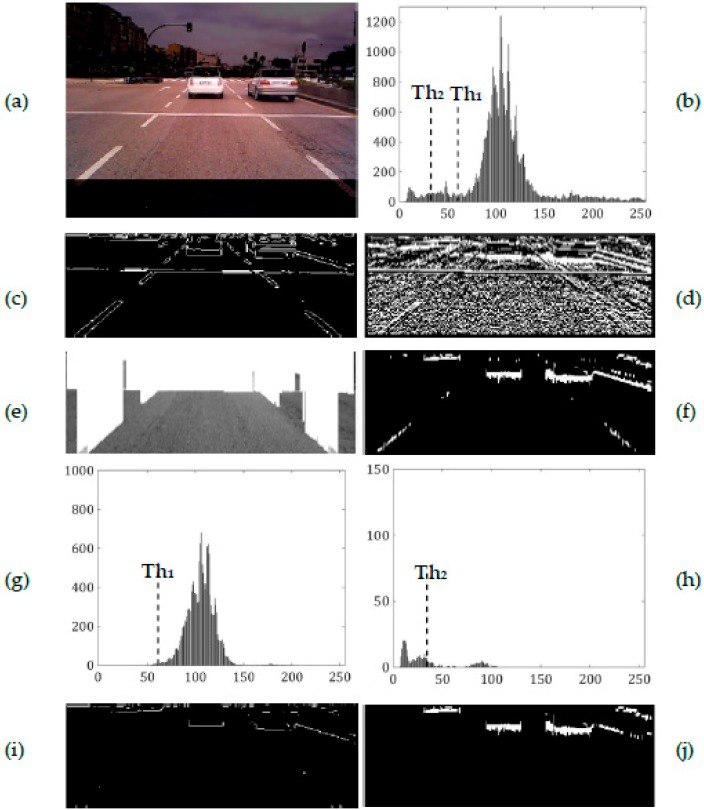
Road scene on cloudy day. (**a**) Searched image area; (**b**) its greyscale histogram. *Left column* (method proposed in [21]): (**c**) edge map, (**e**) free driving road, (**g**) histogram of the free driving road and (**i**) edge map after intensity thresholding. *Righ column* (our method): (**d**) vertical intensity gradients, (**f**) *M*(*x*, *y*) after application of saturation and intensity constraints, (**h**) histogram of set of upper pixels of the gradients and (**j**) *M*(*x*, *y*) after intensity thresholding.

Figure 9a shows the searched image region of a road with vehicles in overcast weather and Figure 9b shows its greyscale histogram with two main intensity peaks. The large peak corresponds to the road pixels and the peak to its left corresponds to pixels of shadows under the vehicles. Pixels of lane markings and regions brighter than the road are on the right of the road peak whereas pixels of asphalt stains and noise as well as kerbs fall between the two main peaks.

The method in [21] establishes the intensity threshold *Th*_1_ as the lower bound intensity of the free driving road (see Figure 9e), giving *m*_1_ = 105.87, *σ*_1_ = 15.27 and *Th*_1_ = 60.04 (see Figure 9g). Edges whose upper region intensity is smaller than *Th*_1_ are candidates of edges of shadow under a vehicle (see Figure 9i). The intensity threshold *Th*_2_ we proposed is established by considering the upper pixels of the gradients remaining in *M*(*x*, *y*) after application of the saturation and intensity difference constraints (see Figure 9f). We obtain *m*_2_ = 34.85, *σ*_2_ = 28.81, and as *σ*_2_ > 1/3·*m*_2_, *Th*_2_ = 34.85 (see Figure 9h). Gradients whose upper pixel intensity is smaller than *Th*_2_ are candidates of gradients of shadow under a vehicle (see Figure 9j).

As stated above, the intensities of upper pixels of the remaining gradients are smaller than that of the road so the threshold *Th*_2_ is smaller than *Th*_1_. Comparing both thresholds (see Figure 9b), *Th*_2_ is closer to the intensity level of shadows under vehicles, thus the thresholding performed with *Th*_2_ is more effective, rejecting a higher number of gradients that are not due to the shadow under a vehicle (see Figure 9i,j). However, this fact becomes more significant in road scenes with lateral shadows or alphalt stains and patches.

**Figure 10:**
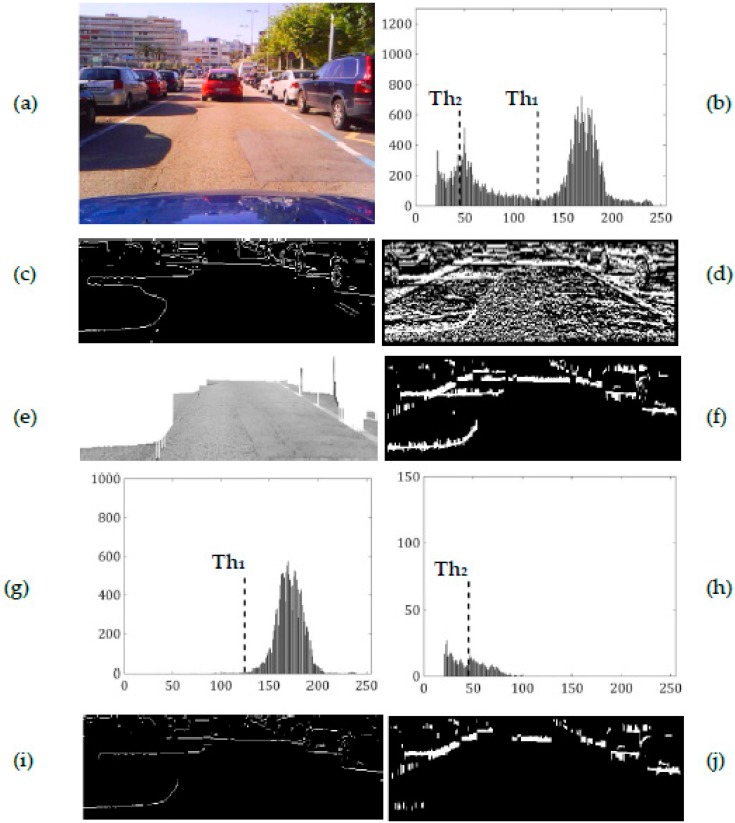
Road scene on a sunny day. (**a**) searched image area, (**b**) its greyscale histogram. *Left column* (method proposed in [21]): (**c**) edge map, (**e**) free driving road, (**g**) histogram of the free driving road and (**i**) edge map after intensity thresholding. *Righ column* (our method): (**d**) vertical intensity gradients, (**f**) *M*(*x*, *y*) after application of saturation and intensity constraints, (**h**) histogram of set of upper pixels of the gradients and (**j**) *M*(*x*, *y*) after intensity thresholding.

Figure 10a shows the searched image region of a road in sunny weather with lateral shadows and Figure 10b shows its greyscale histogram. The intensity level of lateral shadows is higher than that of shadow under vehicles and smaller than that of the road (see Figure 10b). According to [21], from the free driving road pixels we obtain *m*_1_ = 170.87, *σ*_1_ = 15.43 and *Th*_1_ = 124.67 (see Figure 10g). As can be observed in Figure 10b, the lower bound intensity of the road pixels that is *Th*_1_ is much higher than the intensity level of lateral shadows, so the edges of the latter are classified as those due to shadow under a vehicle (see Figure 10i). On the other hand the proposed method provides values of *m*_2_ = 45.61, *σ*_2_ = 18.06 and as *σ*_2_ > 1/3*m*_2_, *Th*_2_ = 45.61 (see Figure 10h). The threshold *Th*_2_ is determined as the mean value of pixels corresponding to shadows under vehicles and lateral shadows, thus it is smaller than the intensity level of most of the latter, rejecting their corresponding gradients from *M*(*x*, *y*) (see Figure 10j). The different performance of *Th*_1_ and *Th*_2_ can be noticed in Figure 10i,j.

Comparing both thresholds (see Figure 10b), *Th*_2_ is closer to the intensity level of shadows under vehicles and thus, more effective than *Th*_1_, rejecting a higher number of gradients that are not due to the shadow under a vehicle including most of those due to lateral shadows.

Using a wide range of test images we verified that the proposed thresholding strategy is more restrictive than that proposed in [21], producing better thresholding results especially on sunny days with lateral shadows and with asphalt patches and stains on the road regardless of the weather.

## 5. Conclusions

This paper presents a new vision-based HG method to detect vehicles ahead in order to avoid front-to-rear collisions. Hypotheses are generated according to the shadow under vehicles and these are reliable cues for vehicle detection in daytime regardless of weather conditions. The proposed strategy overcomes significant difficulties such as the presence of lateral shadows, asphalt stains and traffic markings on the road. The establishment of very conservative thresholds makes the method robust, giving high rates in positive detection on overcast and sunny days. The use of intensity gradients instead of edges to detect shadow boundaries offers three advantages:

1. Gradients ensure the detection of gradual shadow boundaries whose edge detection can easily fail, thus minimizing the number of missed vehicles.
2. Gradients enclose the penumbra of shadows. Thus, pixel properties comparison avoids pixels in penumbra which is partially illuminated by ambient light.
3. The upper pixels of gradients correspond to the bottom of the vehicle making a more accurate framing of its rear especially on sunny days when the sun is in front and the vehicle cast a rear lateral shadow.

Regarding false positives, the rates for overcast and sunny conditions are relatively low for a HG stage. The morphological filter and the consideration only of hypotheses within the ROI strongly contribute to the low rate of false positives which have to be addressed in the hypotheses verification stage. The most frequent error is that vehicles travelling transversely to the ego-vehicle trajectory may satisfy the morphological filter providing hypotheses of vehicles out of the scope of the system.

The proposed method achieves better performance in the intensity thresholding than the method compared, rejecting a higher number of gradients that are not due to the shadow under a vehicle, especially on sunny days with lateral shadows and with asphalt patches and stains on the road regardless of the weather.

As future work, we will first address the incorrect detection of vehicles out of the scope of a system for avoiding front-to-rear collision. Second, we will focus on a hypotheses verification stage in order to develop a complete on-board vehicle detection system. The HV will consist on a learning method based on Support Vector Machine to classify feature vectors extracted from hypotheses to verify whether those hypotheses including false positives, are vehicles.

## Figures and Tables

**Table 1:** Results of Hypotheses Generation.

	Cloudy	Sunny
Total number of frames	7920	5806
Total number of vehicles within the ROI (V)	7303	5115
Total number of hypotheses generated (H)	7830	5555
Positives: Vehicle hypotheses correctly framed (P)	7160	4998
False positives: hypotheses of non-vehicle (FP)	532	449
Vehicle hypotheses incorrectly framed (FNVIF)	138	108
False negatives: vehicles missed (FNVM)	5	9
Positive rate (PR)	98.04%	97.71%
False positive rate (FPR)	6.79%	8.08%
